# The Grave Position at St. George's Hospital

**Published:** 1914-01-17

**Authors:** 


### GRAVE POSITION at ST. GEORGE'S HOSPITAL.

### GOVERNORS ORGANISING. CO-OPERATION INVITED.

INDIGNATION is rising amongst the governors of St. George's Hospital owing to the non-resignation of Mr. West, the treasurer. This feeling is heightened by the report that he and his supporters are leaving no stone unturned to get some sort of a committee of inquiry set up, despite the facts which we published last week and the cruel position in which their action has placed and is placing the President of the hospital. Persistence in this personal policy will leave the large body of independent governors no course open but to secure the holding of a Special Court on February 11, when the whole of the questions involved, their precise nature and effect upon the administration and welfare of St. George's Hospital, must be dealt with. It is a cruel wrong so to permit personal questions to bring lasting discredit upon a great hospital like St. George's. The proceedings referred to, if persisted in, will not be free from danger to the interests of this hospital or to those who may further them.

The governors are organising. We are authorised to state that any governors of St. George's Hospital who may be interested—and which of them is not?—may take a useful part in the defence of St. George's Hospital if they will send their name and address at once to Stuart de la Rue, Esq., Shenley Hill, Shenley, Herts.

### Mr. West and St. George's Hospital.

Reprinted from *Truth* of 14th inst.

"On December 19 the governors of St. George's Hospital, in a court convened by special requisition, rejected by a decisive majority the proposal to appoint Mr. A. W. West secretary-superintendent of the hospital at a stipend of £750 per annum, with quarters. It was to be assumed, and it was so assumed in the last reference to this matter in TRUTH of December 24, that this decisive vote would put an end once and for all to the protracted manœuvres to job Mr. West into a salaried post, which have been going on for the last six months. But Mr. West and his backers do not know when they are beaten. On January 1, to the amazement of all who have taken an interest in the controversy, the announcement appeared in the *Times*." (See THE HOSPITAL, January 10, p. 392.)

### TWENTY-FIVE YEARS' EXPERIENCE OF CHARITY SCANDALS.

"In an experience of charity scandals extending over five-and-twenty years I remember nothing so outrageous as the use that has here been made of Princess Christian's name and position. No 'charges' that could properly be so called, certainly none that would warrant the extraordinary step of the ordering of a special inquiry by the President of the hospital, have been made against the treasurers. As Mr. West was the subject of the proceedings, his record as treasurer may have been criticised by some of the governors who spoke on December 19; but neither in the press reports of the proceedings nor in the memory of persons who were present can I find anything in the nature of 'charges' against either of the treasurers. In the current number of THE HOSPITAL, where the matter is dealt with at considerable length, it is categorically stated that Lord Plymouth's name was never so much as mentioned in the course of the proceedings. The word 'charges' in connection with the financial officers of a charity implies something in the nature of maladministration or malversation of funds; and it is grossly unfair to Lord Plymouth that his name should be published in the *Times* as being the subject of charges which call for independent inquiry by a special tribunal when he has not been criticised nor even referred to. The last sentence in the *Times* announcement implies that Lord Plymouth proposed to resign in consequence of the 'charges' against him. It would be interesting to know what he supposes the charges to be and how he got to know of them. As for Mr. West, he virtually resigned the treasurership many months ago, and all that has taken place since relates to the attempt to find him a salaried post on the staff. This attempt was finally squelched by the governors on December 19, and there, to all intents and purposes, was an end of his official connection with the hospital. In these circumstances to speak of him as 'consenting'—at whose request?—to 'withhold' his resignation is disingenuous and misleading."

### "WHAT HAS OCCURRED IS PERFECTLY OBVIOUS."

"After the decision of the Special Court it was the duty of the House Committee to drop their pertinacious efforts on behalf of Mr. West, and appoint a medical superintendent of the hospital. This most important post has now been vacant for six months, pending the attempt to make a place for Mr. West. Instead of filling it up, Mr. West and his friends have crowned their previous efforts by an attempt to get behind the decision of the governors through the President of the hospital. Princess Christian must have been approached for this purpose by one or other of the irrepressible clique who have been concerned in the previous manœuvres. Her Royal Highness can know nothing of the details of the controversy that has occupied the last six months, beyond what the wirepullers have told her; and she has evidently been misled by them—presumably by this cock-and-bull tale of 'charges' having been made against the treasurers at the governors' meeting. This is a gross offence on the part of those who have been guilty of it. And it is aggravated by the fact that the motive is simply to make a fresh opening for a job which the governors have practically vetoed. A full account of the previous history of the West intrigue will be found in THE

Hospital for January 10, and should be studied by anyone desirous of forming an opinion on the affair."

"Truth's" View of the Motive.

"What is the motive for these reiterated attempts to 'outfit' Mr. West? It is difficult to say, but the important point to be observed is that they have all been engineered and favoured by the medical staff of the hospital and by Mr. West himself, while the opposition has come from the lay committeemen and governors. I think I am right in saying that all the proposals for Mr. West's benefit have emanated from present or former members of the medical staff. Mr. Warrington Haward, who, after the defeat of the proposal to pay the treasurer, moved the appointment of a sub-committee to see how the post of medical superintendent could be manipulated for Mr. West's benefit, is a retired member of the medical staff. The House Committee itself is normally dominated by the medical staff, who, in the natural course of things, are frequently in a majority at its meetings. The Finance Committee which recommended increased emoluments for Mr. West in the post of secretary, which Mr. Haward's sub-committee proposed to jerrymander for his benefit, consisted on this occasion of Mr. Haward, one lay member, and Mr. West himself. That Mr. West should have sat on the Finance Committee at all on this occasion is indecent, but it appears, in addition, to be contrary to one of the laws of the hospital. As the other lay member present dissented from the recommendation of the Finance Committee, it would appear that Mr. West voted with Mr. Haward to make a majority, and The Hospital is informed that he actually wrote the report. When this report was opposed on the House Committee, it was again a layman, Lord Arthur Hill, who took the lead. Finally, at the Special Court, it was the lay governors who squelched the House Committee's proposals, the small minority on Mr. West's side being almost entirely composed of the medical staff."

Important Question of Hospital Administration.

"It follows that we have here at issue one of the most important questions of hospital administration—the question whether in matters of general business directly affecting the welfare of a hospital the medical or the lay element in the management is to be supreme. The solicitude of the medical staff on behalf of Mr. West is quite easy to understand. The treasurer of a hospital—there are two at St. George's, but Mr. West appears to have been the real acting treasurer—is an important and influential personage. Mr. West has taken an active and prominent part in the administration of St. George's for years. He is naturally on friendly terms with the medical staff, past and present; they could not have worked harmoniously together for many years unless he had been so. A fact which may not be without significance is that St. George's has recently been in collision with the King's Hospital Fund over the old question of the appropriation of funds contributed for the medical relief of the poor to the upkeep of the medical school. For what has been done in this respect Mr. West, as treasurer, has been, in part at least, responsible, and his attitude in this matter will naturally have helped to win for him the favour of the medical staff, though the appropriation which was objected to by the King's Fund has now been discontinued."

The Natural Courses.

"It is very natural, therefore, that when Mr. West makes it known, as he has done, that his private affairs do not permit him to continue his work as an honorary treasurer, and suggests or hints that a salary would be acceptable to him, his medical friends in the hospital should do their best to help him. But it is equally natural that the laymen and men of business, with whom the last word in such a matter ought to rest, should take a different view of the situation. They may well think that it is wrong in principle, and would create a vicious precedent, that the administration of a charity should be jerrymandered for no other purpose than to create an income out of the funds for an honorary officer who, from pressure of private circumstances, considers himself in need of it. For doing such a thing there is not in this case even the excuse that the charity is under exceptional obligations to Mr. West. He has no doubt done his duties as treasurer zealously and faithfully; nobody has suggested the reverse. But he has not rendered any brilliant or exceptionally valuable services, such as have been performed gratuitously by some other treasurers of great general hospitals who could easily be named. On the contrary, St. George's is one of the few great hospitals in London whose revenues have not increased substantially in recent years."

A New Point.

"According to my information, it has also happened most unfortunately that, at the very moment when such desperate efforts are being made to utilise the hospital funds for Mr. West's benefit, St. George's very nearly got into a lamentable mess over the proposed sale of the site to Mr. Mallaby-Deeley. But, setting aside any such criticism, and granting that the charity is under the greatest obligations to Mr. West, this does not justify the attempts that have been made to quarter him in future upon its funds. In the administration of a public charity only one object ought to be considered, namely, the good of those for whose benefit the charity exists. If it will conduce to the greater efficiency of St. George's Hospital that it should have a paid instead of an honorary treasurer, or that it shall have a lay as well as a medical superintendent, or that the present secretary should be dismissed and a new one found, any of these things may be rightly done. But to do them in order to oblige a friend, or to assist an honorary officer who needs an increase of income, is utterly wrong. Had the governors of St. George's assented to anything of this kind, they would have been justly accused of perpetrating a job by a misuse of the charitable funds of which they are the guardians, and no apologies that could have been offered for them would have saved the hospital from a scandal which would have seriously impaired its usefulness.

St. George's Hospital Interests First.

"As it is, the charity is now exposed to this very danger by the renewed effort to perpetrate this job in defiance of the governors' veto. Nothing but injury to St. George's can come of these efforts, and no censure can be too strong for those who participate in them. If Mr. West is well advised he will bow to the decision of the governors and accept the severance of his connection with the hospital which his inability to retain the treasurership renders inevitable. By doing this he will relieve the medical staff of the opportunity of contesting further the right of the governors to govern the institution, and at the same time he will enable Princess Christian to retire with dignity from a position into which she ought never to have been led."—*Truth.*

